# Extracellular cholesterol oxidase production by *Streptomyces aegyptia*, *in vitro* anticancer activities against rhabdomyosarcoma, breast cancer cell-lines and *in vivo* apoptosis

**DOI:** 10.1038/s41598-018-20786-3

**Published:** 2018-02-09

**Authors:** Noura El-Ahmady El-Naggar, Hoda M. Soliman, Nancy M. El-Shweihy

**Affiliations:** 1 Department of Bioprocess Development, Genetic Engineering and Biotechnology Research Institute, City of Scientific Research and Technological Applications, Alexandria, Egypt; 2 Department of Botany, Faculty of Science, Mansoura University, Mansoura, Egypt

## Abstract

In recent years, microbial cholesterol oxidases have gained great attention due to its widespread use in medical applications for serum cholesterol determination. *Streptomyces aegyptia* strain NEAE-102 exhibited high level of extracellular cholesterol oxidase production using a minimum medium containing cholesterol as the sole source of carbon. Fifteen variables were screened using Plackett–Burman design for the enhanced cholesterol oxidase production. The most significant variables affecting enzyme production were further optimized by using the face-centered central composite design. The statistical optimization resulted in an overall 4.97-fold increase (15.631 UmL^−1^) in cholesterol oxidase production in the optimized medium as compared with the unoptimized medium before applying Plackett Burman design (3.1 UmL^−1^). The purified cholesterol oxidase was evaluated for its *in vitro* anticancer activities against five human cancer cell lines. The selectivity index values on rhabdomyosarcoma and breast cancer cell lines were 3.26 and 2.56; respectively. The *in vivo* anticancer activity of cholesterol oxidase was evaluated against Ehrlich solid tumor model. Compared with control mice, tumors growth was significantly inhibited in the mice injected with cholesterol oxidase alone, doxorubicin alone and cholesterol oxidase/doxorubicin combination by 60.97%, 72.99% and 97.04%; respectively. These results demonstrated that cholesterol oxidase can be used as a promising natural anticancer drug.

## Introduction

Cardiovascular diseases are related to high blood cholesterol level and colon cancer has been hypothesized to be associated with its degradation products (cholesterol oxides)^1^. Watanabe *et al*.^2^ proposed that the bacterial degradation of cholesterol in foods containing cholesterol may be beneficial to human health. Cholesterol oxidase (EC 1.1.3.6, 3β-hydroxysterol oxidase) is a flavin adenine dinucleotide -dependent enzyme which stimulates cholesterol oxidation using oxygen as an electron acceptor to form cholest-4-en-3-one (cholestenone) and H_2_O_2_^3^.

Because of the strong oxidative activity of cholesterol oxidase toward sterols, it has been used to determine cholesterol and plant sterols in clinical and food specimens^4^. Now, cholesterol oxidase displays a broad range of clinical applications in laboratories such as cholesterol levels quantification in foods and serum which is important in the diagnosis of atherosclerosis, cardiovascular disease and other lipid disorders^5,6^. Also, cholesterol oxidase has been used for the bioconversion of a number of non-steroidal compounds, allylic alcohols and sterols^7^.

Insecticidal activity of bacterial cholesterol oxidase has been reported and it was found to have a potential lethal effect against the boll weevil larvae “*Anthonomus grandis*” which reduces the cotton yields^8^. Bavari *et al*.^9^ have reported the insecticidal effect of cholesterol oxidase when ingested by corn earworm (*Helicoverpa zea*), tobacco budworm (*Heliothis virescens*) and pink bollworm (*Pectinophora gossypiella*). Also, cholesterol oxidase was found to be important for production of the precursors used in the chemical synthesis of steroid hormones^10^. Moreover, Kumari and Kanwar^11^ reported that cholesterol oxidase is implicated in the manifestation of Alzheimer’s disease, HIV and tuberculosis. Recently, the antifungal antibiotic called polyene macrolide pimaricin which used in the food industry as mould inhibitor was biosynthesized by cholesterol oxidase from *Streptomyces natalensis*^12^. The microbial cholesterol oxidase from *Bordetella* sp. when used in lung cancer treatment “both *in vitro* and *in vivo* led to irreversible cell apoptosis by reducing cholesterol content and rising reactive oxygen species level. For this reason, cholesterol oxidase may be a promising enzyme for a novel anti-tumor therapy” as reported by Liu *et al*.^13^.

Many microorganisms were demonstrated to produce cholesterol oxidase including *Mycobacterium* species and *Nocardia rhodochrous*^14^ which produce cholesterol oxidases as an intrinsic membrane bound enzymes located on the outside of the cell, whereas *Streptomyces fradiae*^15^, *Streptomyces violascens*, *Streptomyces parvus*^16^, *Streptoverticilium cholesterolicum*^17^, *Rhodococcus erythropolis* and *Rhodococcus equi, Arthrobacter simplex* and *Shizophylum commune*^18^ produce extracellular cholesterol oxidase in the broth filtrate.

Cholesterol oxidases from *Streptomyces* species have been reported to have more desirable properties than those from other microorganisms. “Lolekha and Jantaveesirirat^19^ compared performance of cholesterol oxidase from three sources (*Sreptomyces* sp., *Nocardia* and *Pseudornonas*) for determination of serum cholesterol by binding the enzyme with peroxidase. They reported that the cholesterol oxidase isolated from *Streptomyces* sp. was superior to those produced by *Pseudomonas* sp. and *Nocardia* due to lower cost of production and better stability”. Furthermore, the reagent containing cholesterol oxidase isolated from *Streptomyces* had the longest shelf-life and performed superior performance as compared with the reagent contained cholesterol oxidase produced by *Pseudomonas* or *Nocardia*. Cholesterol oxidase currently used in organic synthesis because “it is stable in water immiscible solvents at high concentrations and shows excellent regio, stereio and enantioselectivity in the oxidation of non-steroidal compounds”. Many applications have been done using cholesterol oxidase produced by *Streptomyces* species; it appears to be a promising culture to produce cholesterol oxidase commercially^20^.

This study aimed to optimize the cultural conditions for a higher production of cholesterol oxidase by *Streptomyces aegyptia* NEAE-102 including the screening of the variables influencing cholesterol oxidase production using Plackett-Burman design, determination of the optimum levels of the significant factors that influence the production of cholesterol oxidase using face-centered central composite design and to assess the *in vitro* anticancer activities of cholesterol oxidase treatment on various human cancer cell lines. Moreover, to assess the *in vivo* anticancer activities of cholesterol oxidase alone or in combination with doxorubicin (Dox) against Ehrlich solid tumor model.

## Results and Discussion

### Statistical screening of factors influencing cholesterol oxidase production by *Streptomyces aegyptia* strain NEAE 102 using Plackett-Burman design

Firstly, Plackett-Burman statistical design was used to assess the impacts of carbon sources (glucose, cholesterol, starch), energy source (K_2_HPO_4_), metals (NaCl, MgSO_4_.7H_2_O, FeSO_4_. 7H_2_O), nitrogen sources (yeast extract, peptone, (NH_4_)_2_SO_4_), in addition to the physical parameters (pH, medium volume, time of incubation, temperature, inoculum size) on cholesterol oxidase production by the selected strain. Secondly, a face centered central composite design was used for optimization of significant variables and studying their interactions on cholesterol oxidase production.

Compared with other strategies for designing a growth medium, the Plackett-Burman design is uncomplicated and quick method for screening a large number of variables in one experiment to evaluate the significant variables affecting the cultural requirements and the production of enzyme in fermentation broth^21,22^. It was carried out by performing 20 runs for identifying the key variables that increase cholesterol oxidase production by the selected strain. Table 1 illustrates the experimental design and the fifteen independent variables as well as levels of each variable used in the experimental design.

**Table 1:** Two levels of the independent variables which selected for production of cholesterol oxidase by *Streptomyces aegyptia* NEAE 102 using Plackett–Burman design.

Variable code	Variables	Levels	
		Low actual (−1)	High actual (1)
A	Cholesterol (g/L)	1	2
B	Starch (g/L)	7	10
C	Glucose (g/L)	10	15
D	Yeast extract (g/L)	4	6
E	Peptone (g/L)	3	5
F	(NH_4_)_2_SO_4_ (g/L)	6	8
G	K_2_HPO_4_ (g/L)	0.5	1
H	NaCl (g/L)	0.5	1
J	MgSO_4_.7H_2_O (g/L)	0.2	0.5
K	FeSO_4_.7H_2_O (g/L)	0.0	0.02
L	Temperature (°C)	30	37
M	Incubation time (days)	5	7
N	Inoculum size (%, v/v)	2	4
O	Medium volume (mL/ 250 mL conical flask)	50	100
P	pH	7	9

Table 2 represents the effect of fifteen independent factors with coded levels on the cholesterol oxidase production using twenty-trials according to Plackett-Burman design. Results of different trials show a variation in findings from 0.837 to 7.861 UmL^−1^. This change reflects the importance of medium optimization to achieve high enzyme production. The maximum cholesterol oxidase production (7.861 UmL^−1^) was achieved in the sixteenth run, while the minimum cholesterol oxidase production (0.837 UmL^−1^) was observed in the third run.

**Table 2:** The effect of fifteen independent factors with coded levels on cholesterol oxidase activity using twenty-trials according to “Plackett–Burman experimental design”.

Std	Run no.	Coded values of the independent factors																			Cholesterol oxidase activity (UmL^−1^)		Residuals
		A	B	C	D	E	F	G	H	J	K	L	M	N	O	P	Dummy 1	Dummy 2	Dummy 3	Dummy 4	Actual value	Predicted value	
4	1	1	1	−1	1	1	−1	−1	1	1	1	1	−1	1	−1	1	−1	−1	−1	−1	3.861	4.051	−0.190
13	2	1	−1	1	−1	1	−1	−1	−1	−1	1	1	−1	1	1	−1	−1	1	1	1	4.775	4.776	−0.001
15	3	1	1	1	−1	1	−1	1	−1	−1	−1	−1	1	1	−1	1	1	−1	−1	1	0.837	0.715	0.123
7	4	−1	−1	−1	1	1	−1	1	1	−1	−1	1	1	1	1	−1	1	−1	1	−1	4.174	4.242	−0.069
6	5	−1	−1	1	1	−1	1	1	−1	−1	1	1	1	1	−1	1	−1	1	−1	−1	1.412	1.465	−0.053
16	6	1	1	1	1	−1	1	−1	1	−1	−1	−1	−1	1	1	−1	1	1	−1	−1	4.951	4.813	0.138
12	7	−1	1	−1	1	−1	−1	−1	−1	1	1	−1	1	1	−1	−1	1	1	1	1	2.670	2.480	0.190
17	8	−1	1	1	1	1	−1	1	−1	1	−1	−1	−1	−1	1	1	−1	1	1	−1	2.716	2.839	−0.123
18	9	−1	−1	1	1	1	1	−1	1	−1	1	−1	−1	−1	−1	1	1	−1	1	1	3.496	3.443	0.053
8	10	−1	−1	−1	−1	1	1	−1	1	1	−1	−1	1	1	1	1	−1	1	−1	1	2.872	2.804	0.069
14	11	1	1	−1	1	−1	1	−1	−1	−1	−1	1	1	-1	1	1	−1	−1	1	1	2.556	2.693	−0.138
5	12	−1	1	1	−1	1	1	−1	−1	1	1	1	1	−1	1	−1	1	−1	−1	−1	3.799	3.798	0.001
9	13	1	−1	−1	−1	−1	1	1	−1	1	1	−1	−1	1	1	1	1	−1	1	−1	4.825	4.894	−0.069
1	14	1	1	−1	−1	1	1	1	1	−1	1	−1	1	−1	−1	−1	−1	1	1	−1	5.087	5.209	−0.123
2	15	−1	1	1	−1	−1	1	1	1	1	−1	1	−1	1	−1	−1	−1	−1	1	1	4.808	4.946	−0.138
19	16	1	−1	−1	1	1	1	1	−1	1	−1	1	−1	−1	−1	−1	1	1	−1	1	7.861	7.601	0.259
10	17	−1	1	−1	−1	−1	−1	1	1	−1	1	1	−1	−1	1	1	1	1	−1	1	3.794	3.535	0.259
11	18	1	−1	1	−1	−1	−1	−1	1	1	−1	1	1	−1	−1	1	1	1	1	−1	4.038	3.970	0.068
3	19	1	−1	1	1	−1	−1	1	1	1	1	−1	1	−1	1	−1	−1	−1	−1	1	6.431	6.499	−0.068
20	20	−1	−1	−1	−1	−1	−1	−1	−1	−1	−1	−1	−1	−1	−1	−1	−1	−1	−1	−1	4.019	4.209	−0.190

“Two level design, each variable is tested at a low “−1” and high “+1” value.”

**Figure 1:**
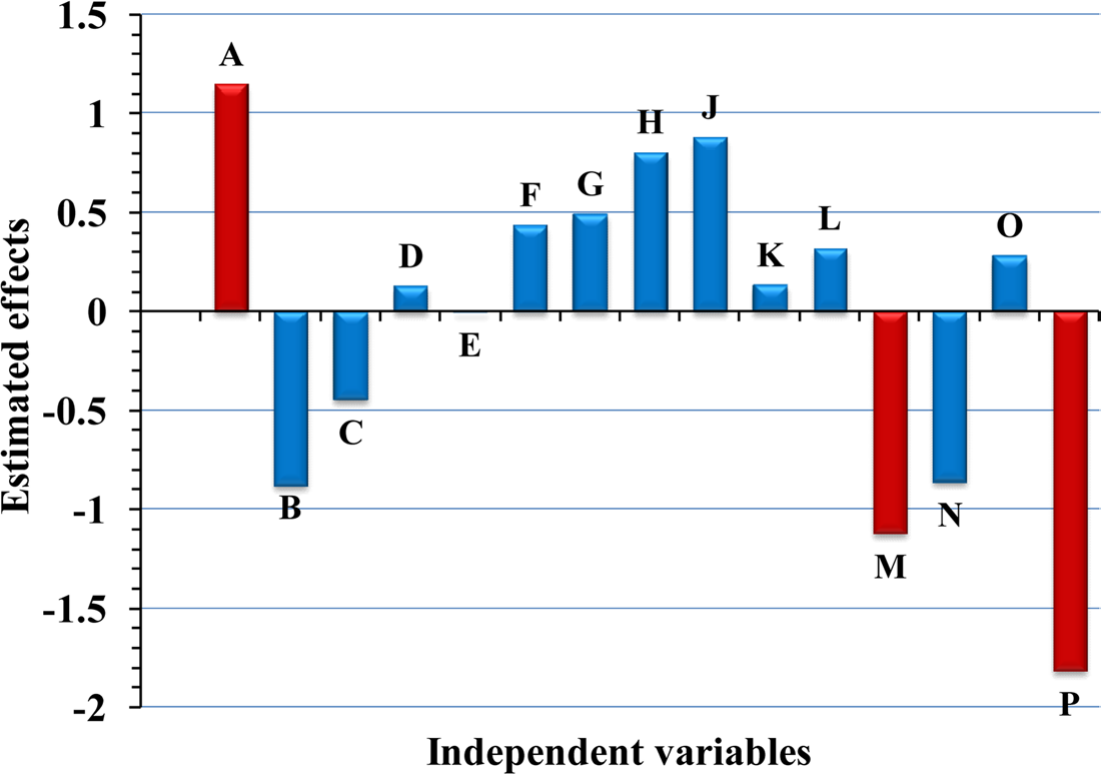
Estimated effects of independent variables on the enzyme production by the selected strain “The red color represents the most significant independent variables affecting enzyme production”.

To determine the correlations between the independent factors and production of cholesterol oxidase, a multiple-regression mathematical model was used. Statistical analysis was achieved and summarized in Tables 3, 4. Table 3 and Fig. 1 illustrate the estimated effect of the tested parameters on the enzyme production. Main effect enables the estimation of the influence of each factor on the enzyme production. Both large positive or negative effects indicate that a variable has a large impact on the production, while the factor has little effect or considered as uneffective with value close to zero. From the main effect results, we can find that nine of the fifteen variables named cholesterol, yeast extract, (NH_4_)_2_SO_4_, K_2_HPO_4_, NaCl, MgSO_4_, FeSO_4_, temperature and the volume of medium have a positive influence on the production of enzyme, where the other six variables namely glucose, peptone, inoculum size, starch, pH and incubation time negatively affect the cholesterol oxidase production. “Variables with positive impacts on the production of cholesterol oxidase have been used at high level, while the variables that have a negative effect are kept at low level for further optimization by face-centered central composite design”.

**Table 3:** Statistical analysis of Plackett–Burman design showing regression coefficients, estimated effect and % of contribution of the tested factors on the enzyme production by *Streptomyces aegyptia* NEAE-102.

Term	Coefficient	Effect	% Contribution
Intercept	3.949		
A	0.573	1.146	11.76
B	−0.441	−0.882	9.06
C	−0.223	−0.446	4.58
D	0.064	0.127	1.31
E	−0.001	−0.002	0.02
F	0.218	0.435	4.48
G	0.245	0.491	5.03
H	0.402	0.804	8.25
J	0.439	0.878	9.01
K	0.066	0.132	1.36
L	0.159	0.317	3.26
M	−0.562	−1.123	11.54
N	−0.431	−0.861	8.85
O	0.140	0.280	2.87
P	−0.908	−1.817	18.64

**Table 4:** Regression statistics and analysis of variance (ANOVA).

Item	Sum of squares	*Df*	Mean square	*F-*value	*t -*Stat	*P*-value	Confidence level (%)
Model	48.274	15	3.218	34.430	57.765	0.0018*	99.82
A	6.568	1	6.568	70.269	8.383	0.0011*	99.89
B	3.892	1	3.892	41.643	−6.453	0.0030*	99.7
C	0.993	1	0.993	10.621	−3.259	0.0311*	96.89
D	0.081	1	0.081	0.863	0.929	0.4055	59.45
E	0.000	1	0.000	0.000	−0.018	0.9865	1.35
F	0.946	1	0.946	10.126	3.182	0.0335*	96.65
G	1.205	1	1.205	12.891	3.590	0.0230*	97.7
H	3.234	1	3.234	34.595	5.882	0.0042*	99.58
J	3.856	1	3.856	41.252	6.423	0.0030*	99.7
K	0.087	1	0.087	0.929	0.964	0.3898	61.02
L	0.503	1	0.503	5.385	2.321	0.0811	91.89
M	6.307	1	6.307	67.477	−8.214	0.0012*	99.88
N	3.709	1	3.709	39.676	−6.299	0.0032*	99.68
O	0.393	1	0.393	4.206	2.051	0.1096	89.04
P	16.500	1	16.500	176.521	−13.286	0.0002*	99.98
Residual	0.374	4	0.093				
Cor Total	48.648	19					
Std. Dev.	0.3057		R-Squared		0.9923		
Mean	3.9491		Adj R-Squared		0.9635		
C.V.%	7.7419		Pred R-Squared		0.8079		
PRESS	9.3473		Adeq Precision		25.1830		

“*Significant values, *df*: Degree of freedom, *F*: Fishers’s function, *P*: Level of significance, C.V.: Coefficient of variation, PRESS: the predicted residual sum of squares. The correlation coefficient (R)= 0.9961.”

**Figure 2:**
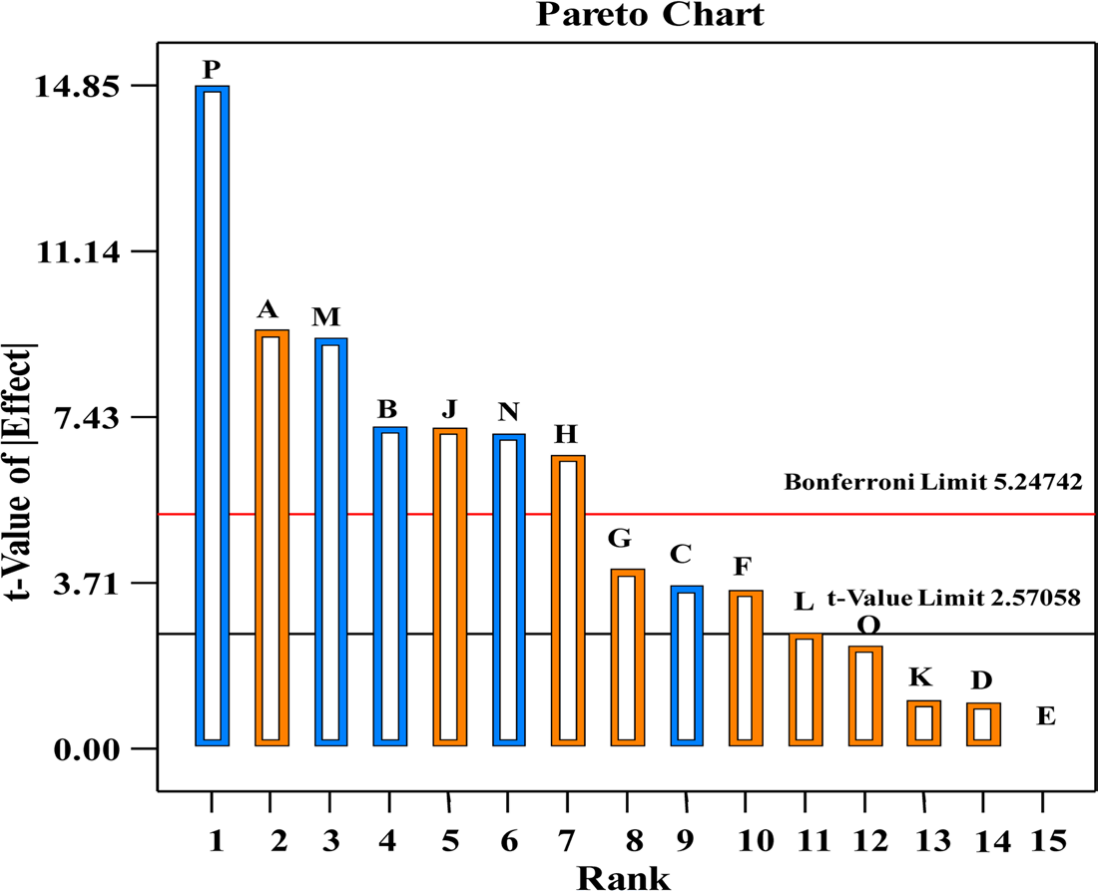
Pareto chart for Plackett-Burman design rationalizing the order and the impact of each factor on the enzyme production by the selected strain (orange and blue color represent the positive and negative effects; respectively).

The contribution percentage of each factor are presented in Table 3; pH, cholesterol and incubation time are the most contributing components with 18.64, 11.76, and 11.54%; respectively. As shown in Fig. 2, the Pareto chart offers an easy way to view the results obtained by Plackett-Burman design, it illustrates the significance order of the factors affecting cholesterol oxidase production. “It shows the absolute values of the effects, and draws a reference line on the chart. Any effect that extends past this reference line is potentially important”. Pareto chart in design expert version 7.0 reproduce the relation between *t*-value (effect) vs. ranks. Among the 15 assigned variables, pH was the most significant variable affecting cholesterol oxidase production at 99.98% confidence followed by cholesterol at 99.89% confidence then incubation time at 99.88% confidence.

To validate the obtained results of the experimental design, analysis of variance (ANOVA) for cholesterol oxidase production was performed and confidence level, *F*-value, sum of square, *P-* value, *t*-value (*t*-Stat) and the mean square are presented in Table 4. The *P*- value is the probability which serves as a tool for testing the significance of the model and each variable. “A low *P*-value indicates a real or significant effect. The values of *P* < 0.05 indicate model terms are significant. The significance of each variable was determined by applying the Student’s *t*-test”. The *P*-value of 0.0018 and the model *F*-value of 34.43 indicate that this model is significant.

The ANOVA analysis displayed that, pH (p), with *t*-value of −13.286, *P* -value of 0.0002 and confidence level of 99.98% was found to be more significant variable, followed by cholesterol (A) (probability value of 0.0011, *t*-value 8.383, and confidence level 99.89%), then incubation time (M) (*t*-value −8.214, probability value of 0.0012 and confidence level 99.88%), the lower probability values indicate significant variables influencing the production of cholesterol oxidase. Furthermore, it was obvious that among the three factors, only cholesterol exerted a positive effect, whereas the other factors (pH and incubation time) exerted negative effects on cholesterol oxidase production, which means that the increase in cholesterol concentration and a decrease in pH and incubation time could exert positive impact on cholesterol oxidase production. Screened significant variables (cholesterol, starch, glucose, (NH_4_)_2_SO_4_, K_2_HPO_4_, NaCl, MgSO_4_.7H_2_O, inoculum size, time of incubation and pH) strongly affects the cholesterol oxidase production, while temperature, medium volume, yeast extract, peptone and FeSO_4_.7H_2_O, of no significant influence on the enzyme production.

A good correlation between the experimental and predicted values was indicated by a higher correlation coefficient (R = 0.9961) value. “The coefficient of determination (R^2^) value explains a measure of how much variability in the observed response values can be illustrated by the experimental factors. The value of R^2^ is always between 0, 1. The closer the R^2^ to 1, the stronger is the model and the better predicted response”^23^. The coefficient of determination value (R^2^ = 0.9923) showed that 99.23% of the variations in the enzyme production can be demonstrated by the independent factors and only 0.77% of the variations are not demonstrated by these factors. Also, a very high adjusted coefficient of determination value (Adj. R^2^ = 0.9635) indicates the highly significance of the model^24^. The “Pred R-Squared” value of 0.8079 is close to the “adj R-Squared” value of 0.9635. This elucidates a good correlation between the predicted and experimental values of cholesterol oxidase production. “Adeq Precision estimates the signal to noise ratio. A ratio greater than 4 is desirable”. Ratio of 25.183 indicates an adequate signal. “The coefficient of variation% (C.V.%) is a measure of residual variation of the data relative to the size of the mean. Usually, the higher the value of C.V., the lower is the reliability of experiment”. In this study, the C.V.% value (7.7419%) is very low which gives a greater precision of the performed experiments. “The predicted residual sum of squares (PRESS) is a measure of how well the model fits each point in the design. The smaller the PRESS statistics, the better the model fits the data points”. The PRESS value is 9.3473. In this experiment, the model gives values of mean and standard deviation of 3.9491 and 0.3057; respectively.

A first order polynomial equation was applied to represent the optimum cholesterol oxidase production as a function of the independent factors. By ignoring the insignificant terms, the following equation of regression in terms of coded factors was obtained:

1 $$\begin{array}{rcl}{{\bf{Y}}}_{({\bf{Cholesterol}}{\bf{oxidase}}{\bf{activity}})} & = & +3.949+0.573{\rm{A}}-0.441{\rm{B}}-0.223{\rm{C}}+0.218{\rm{F}}\\  &  & +\,0.245{\rm{G}}+0.402{\rm{H}}+0.439{\rm{J}}-0.562{\rm{M}}-0.431{\rm{N}}-0.908{\rm{P}}\end{array}$$

Where Y is the response and “A, B, C, F, G, H, J, M, N and P” are cholesterol, starch, glucose, (NH_4_)_2_SO_4_, K_2_HPO_4_, NaCl, MgSO_4_.7H_2_O, time of incubation, inoculum size and pH; respectively.

Based on the main effect (Table 3) and the calculated *t*-values (Table 4), cholesterol concentration (A), incubation time (M) and pH (P) were chosen to further optimize by FCCD, as those variables represented the more significant effect on cholesterol oxidase production.

### Adequacy of the model

**Figure 3:**
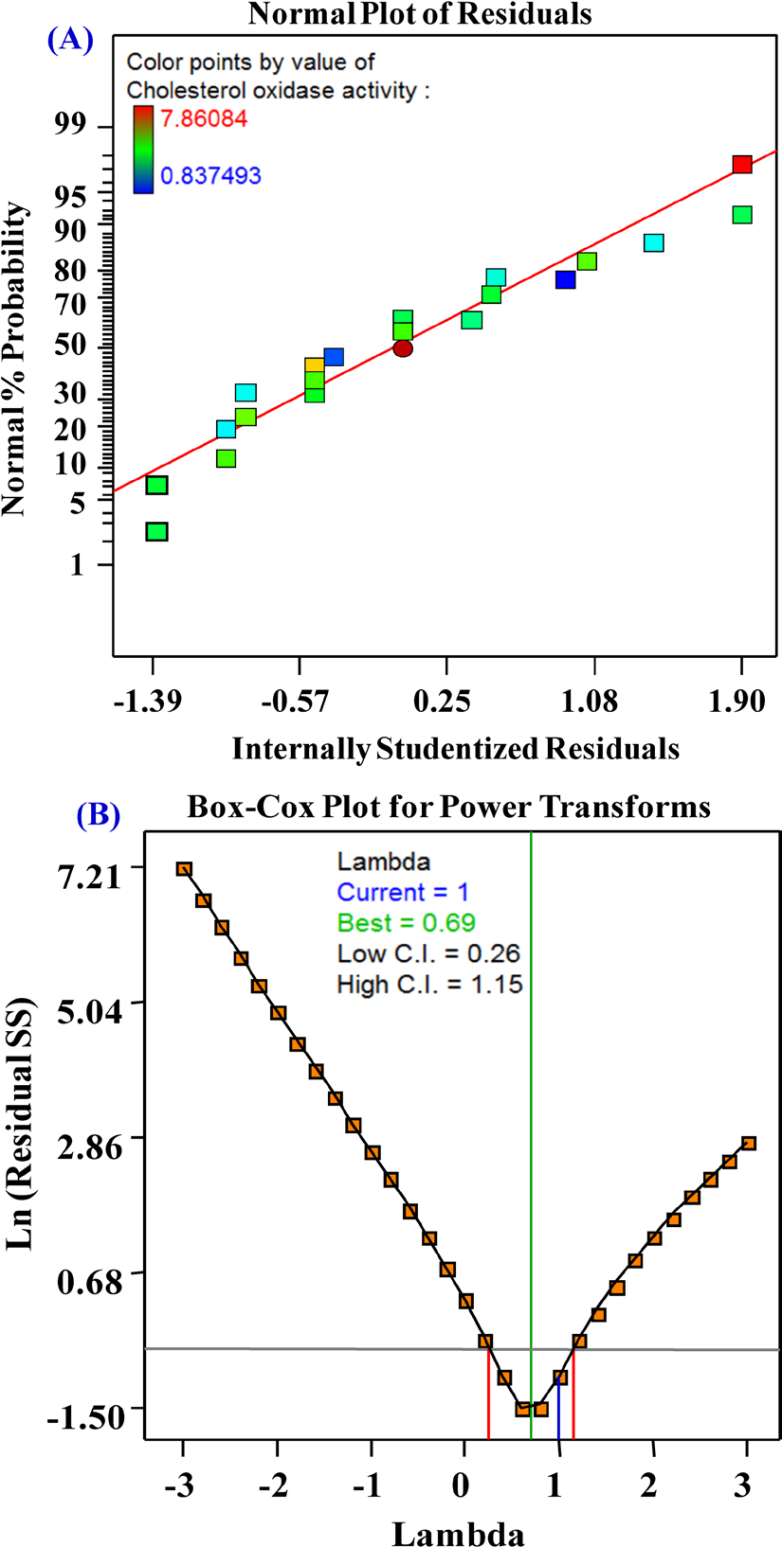
(**A**) Normal probability plot; (**B**) Box-Cox plot for model power transformation.

The normal probability plot (NPP) is given in Fig. 3A. “The normal probability plot of the residuals is an important diagnostic tool to reveal and demonstrate the systematic departures from the normality”^25^. NPP of internally studentized residuals illustrates the points near the diagonal line which means that the residuals are normally distributed and this model was well fitted with the experimental results. Box–Cox plot represents a potential best practice for selecting a better power transformation to further improve the model. As observed from Fig. 3B, the blue line indicates the current transformation (Lambda = 1) and the best lambda value indicated by the green line (Lambda = 0.69), while the lines that are red in color indicate a minimum (0.26) and maximum (1.15) 95% confidence interval values. So, this model needs no transformation, as current value of confidence interval (λ = 1) is very close to the value of model design (best = 0.69) and the model is in the optimal zone since the blue line falls within the red lines. So that the model is well fit to the obtained experimental data.

In the confirmatory experiment, for evaluation of Plackett-Burman design accuracy, production medium of the following formula (g/L): cholesterol 2; starch 7; yeast extract 6; glucose 10; peptone 3; (NH_4_)_2_SO_4_ 8; K_2_HPO_4_ 1; NaCl 1; MgSO_4_.7H_2_O 0.5; FeSO_4_.7H_2_O 0.02 with medium volume of 100 mL/ 250 mL conical flask and pH 7.0; inoculated with 2% (v/v) inoculum size and incubated at 37 **°**C for 5 days, gives 2.5 times fold increase in cholesterol oxidase activity of 7.75 UmL^−1^ in comparison of the obtained activity before employing Plackett-Burman design (3.1 UmL^−1^).

An enhanced cholesterol oxidase production was reported by using compounds like yeast extract, cholesterol^15^, potato starch, malt extract and peptone^20^ as substrates. Cholesterol is largely metabolized by microorganisms as a source of energy and carbon^26^. Different microorganisms like *Brevibacterium*, *Arthrobacter*, *Mycobacterium*, *Nocardia*, *Streptomyces* and *Corynebacterium* have the capability of degrading cholesterol. The initial step of cholesterol degradation by microorganisms is the oxidation of the 3β- hydroxyl group by cholesterol oxidase. Consequently, cholesterol degrading microbes are commonly considered to be cholesterol oxidases producer^27^. Yehia *et al*.^28^ reported that the decomposition of cholesterol by the tested bacterial isolates was affected by cholesterol concentration in the cultural medium. The highest percentage of cholesterol breakdown (80.2%) by the *Enterococcus hirae* was displayed when 1 g/L cholesterol was added and the highest cholesterol breakdown by *Rhodococcus erythropolis*^29^ and *Streptomyces fradiae*^15^ was accomplished at 2 g/L cholesterol.

The pH value of the cultural medium is extremely crucial for growth of microorganisms, their metabolic characteristics and also for the biosynthesis of metabolites. The hydrogen ion concentration can have a direct impact on the cell or may cause an indirect effect on the cell by changing the degree of dissociation of the constituents of the medium^30^. Yehia *et al*.^28^ found that the change in the cultural medium pH significantly affect the maximal physiological performance of the cells, the transfer of different nutrients through the membrane of the cell and the breakdown of cholesterol. Yazdi *et al*.^15^ reported that the best pH value for cholesterol breakdown by *Streptomyces fradiae* was 7.2, while Sojo *et al*.^29^ found that pH 6.75 was the best for cholesterol breakdown by *Rhodococcus erythropolis* ATCC 25544. In addition, Yehia *et al*.^28^ reported that the highest pH value needed for maximum decomposition of cholesterol and growth of *Enterococcus hirae* in liquid medium was pH 7.0. A maximal pH 8 was reported for a new alkaline *Streptomyces* species which isolated from the east African soda lakes^31^. Also, Solingen *et al*.^31^ highlighted the effect of alkaline pH on the growth and adaptation of *Streptomyces* species.

The incubation period is an important factor that must be considered. Since the production of cholesterol decomposing enzyme could be subjected to alteration with the long incubation period. For example, the maximum decomposition of cholesterol by non-irradiated and Nd-YAG irradiated bacteria was detected at the end of the sixth day. The percentage of cholesterol decomposition, on the sixth day, amounted 82.8 in the case of irradiated *Streptomyces fradiae* as compared to 66.9% in the case of non-irradiated one. Generally, the decomposition of cholesterol, at any incubation time, by the irradiated *Streptomyces fradiae* significantly exceeded that recorded by non-irradiated bacterium. Several investigators indicated different incubation periods for different microorganisms. Abo-El-Khair^32^ reported that the cholesterol decomposing activity of *Mycobacterium fortuitum* increased rapidly during the first three days of incubation followed by insignificant variations with extension of incubation up to 10 days. It can be mentioned here that the process of cholesterol decomposition by microorganisms occurs during 6 days for *Streptomyces* spp.^33^ and 43 hr. for the most active strains of actinomycetes^34^. Watanabe *et al*.^2^ reported that most *Rhodococcus* strains complete the cholesterol degradation during 3–7 days of incubation period. A maximum production obtained by *Bacillus cereus* strain KAVK4 at 32 hours of incubation period was 1.67 UmL^−1^. Niwas *et al*.^35^ reported that the period of incubation has taken an important role in cholesterol oxidase enzyme production by *Streptomyces* sp. The culture grew exponentially up to 56 h and then entered into a stationary phase that was continued for up to 96 h. In the exponential phase the enzyme production was initiated, at stationary phase of the growth the enzyme production reached to the maximum and then declined as a result of the consumption of carbon source and other nutrients. *Streptomyces lavendulae*^22^ and *Streptomyces parvus*^17^ have been also outlined for their optimal production of cholesterol oxidase enzyme at the stationary phase of growth.

### Optimization of fermentation process using face-centered central composite design (FCCD)

Experiment of Plackett -Burman design showed that, cholesterol concentration (X_1_), time of incubation (X_2_) and the value of pH (X_3_) influenced the cholesterol oxidase production effectively. So, FCCD was employed to determine the optimum levels of these variables which give maximum production of cholesterol oxidase and their interactions with each other. “Variables with positive impacts on the production of cholesterol oxidase have been used at high level, while the variables that have a negative effect are kept at low level for further optimization by FCCD”. However, among the variables that negatively affect cholesterol oxidase production, only inoculum size was kept in all trials at its low level because it was associated with the composition of the growth medium and can’t be deleted. Starch and glucose that have negative influences on the production of cholesterol oxidase were omitted from medium composition. On the other hand, peptone, yeast extract and FeSO_4_.7H_2_O, which had no significant effects on cholesterol oxidase production, also omitted from the subsequent experiments. The effect of cholesterol concentration (X_1_), time of incubation (X_2_) and the value of pH (X_3_) and their interactions on cholesterol oxidase production were studied by FCCD at three levels (−1, 0, 1) using 20 runs and the results are presented in Table 5.

**Table 5:** FCCD showing cholesterol oxidase production by the selected strain as influenced by the three most significant variables along with the predicted values and residuals.

Std	Run	Type	Variables			Cholesterol oxidase activity (U mL^−1^)			Residuals
			X_1_	X_2_	X_3_	Experimental		Predicted	
5	1	Factorial	−1	−1	1	9.065		8.875	0.190
6	2	Factorial	1	−1	1	12.663		12.649	0.014
14	3	Axial	0	0	1	15.411		15.459	−0.048
3	4	Factorial	−1	1	−1	10.172		10.022	0.150
18	5	Center	0	0	0	14.710		14.940	−0.230
7	6	Factorial	−1	1	1	7.939		8.084	−0.145
12	7	Axial	0	1	0	14.808		14.777	0.031
11	8	Axial	0	−1	0	13.349		14.037	−0.688
4	9	Factorial	1	1	−1	8.823		8.848	−0.026
16	10	Center	0	0	0	15.390		14.940	0.450
1	11	Factorial	−1	−1	−1	6.193		6.018	0.175
2	12	Factorial	1	−1	−1	6.885		6.576	0.309
15	13	Center	0	0	0	15.074		14.940	0.134
8	14	Factorial	1	1	1	10.116		10.127	−0.011
10	15	Axial	1	0	0	10.310		10.597	−0.287
9	16	Axial	−1	0	0	8.926		9.297	−0.370
20	17	Center	0	0	0	15.074		14.940	0.134
19	18	Center	0	0	0	15.631		14.940	0.691
13	19	Axial	0	0	−1	12.781		13.391	−0.609
17	20	Center	0	0	0	15.074		14.940	0.134
**Variable**			**Variable code**		**Coded and actual levels**				
					−**1**	**0**	**1**		
Cholesterol (g/L)			X_1_		2	3	5		
Incubation time (days)			X_2_		4	5	6		
pH			X_3_		5	6	7		

On the basis of experimentaly obtained data; the activity of cholesterol oxidase extended from 6.193 to 15.631 UmL^−1^. The maximum enzyme activity was observed in the run number 18 (center point) with a value of 15.631 UmL^−1^, where cholesterol concentration 3 g/L, incubation time 5 days and pH 6 were used. Whereas the minimum activity of cholesterol oxidase was obtained in the run number 11 with a value of 6.193 UmL^−1^, where cholesterol concentration 2 g/L, incubation time 4 days and pH 5 were used. In Table 5 each experimental value of cholesterol oxidase activity was in good correlation with the model predictable value.

In the current study, the maximum enzyme activity (yield) of cholesterol oxidase of *Streptomyces aegyptia* NEAE-102 was 15.631 UmL^−1^, which was higher than the activity of cholesterol oxidase enzyme of *Streptomyces lavendulae* (2 UmL^−^^1^)^36^, *Rhodococcus equi* no. 23 (0·24 UmL^−1^)^37^, *Streptomyces fradiae* (0·03 UmL^−1^)^38^, *Bacillus cereus* (1.67 UmL^−1^)^39^, *Streptomyces* sp. (6.2 UmL^−1^)^35^, *Brevibacterium* sp. (1.483 UmL^−1^)^40^, *Streptomyces* A, and B (2.44 and 2.25 UmL^−1^; respectively)^41^ and *Micrococcus* sp. (3.68 UmL^−1^)^42^.

### Multiple regression analysis and ANOVA

The obtained data were subjected to regression analysis and analysis of variance (ANOVA) using the Design Expert 7.0 software, and the generated data are represented in Tables 6–8. The coefficient of determination (R^2^) checks the goodness of fit of the model. In the present investigation, the obtained coefficient of determination (R^2^) value (0.9895) (Table 6) reflected that 98.95% of the variations in the enzyme production can be explained by the independent factors and only 1.05% of the variations are not explained by these factors. According to Chen *et al*.^43^, “a regression model is considered highly correlated when the R^2^ value is higher than 0.9”. The adjusted determination coefficient (Adj R^2^) found to be 0.9801 which confirm the model significance. The values of the “Pred R-Squared and the “Adj R-Squared” were found to be 0.9494 and 0.9801, respectively, which elucidates a very good fit between the experimentally obtained results and the theoretically predicted values by the model^44^ and implied that the model is reliable for cholesterol oxidase production in the present study. Furthermore, Adeq Precision value was very high (29.4015) which indicates that the model can be used to navigate the design space. The lower value of coefficient of variation % (3.810) indicated a better precision and reliability of the experimental performance^45^. The obtained PRESS value is 9.971, the model gives values of mean and standard deviation of 11.920 and 0.454; respectively (Table 6).

**Table 6:** Regression statistics of FCCD, regression coefficients of second order polynomial model for cholesterol oxidase production optimization.

Factor	Coefficient estimate	Standard error	95% CI Low	95% CI High
Intercept	14.940	0.156	14.592	15.287
X_1_ - (Cholesterol, g/L)	0.650	0.144	0.330	0.970
X_2_ - (Incubation time, days)	0.370	0.144	0.050	0.690
X_3_ - (pH)	1.034	0.144	0.714	1.354
X_1_ X_2_	−0.433	0.161	−0.790	−0.075
X_1_ X_3_	0.804	0.161	0.446	1.162
X_2_ X_3_	−1.199	0.161	−1.556	−0.841
X_1_^2^	−4.992	0.274	−5.603	−4.382
X_2_^2^	−0.532	0.274	−1.143	0.078
X_3_^2^	−0.515	0.274	−1.125	0.095
Std. Dev.	0.454	R-Squared		0.9895
Mean	11.920	Adj R-Squared		0.9801
C.V.%	3.810	Pred R-Squared		0.9494
PRESS	9.971	Adeq Precision		29.4015

C.V: Coefficient of variation.

**Table 7:** Analysis of variance (ANOVA) for the quadratic regression model obtained from FCCD.

Source	Sum of Squares	*Df*	Mean Square	*F-*value	*P-*value *P*rob > *F*
Model	194.84	9	21.65	104.99	<0.0001*
X_1_ - (Cholesterol, g/L)	4.23	1	4.23	20.50	0.0011*
X_2_ - (Incubation time, days)	1.37	1	1.37	6.65	0.0275*
X_3_ - (pH)	10.69	1	10.69	51.85	<0.0001*
X_1_ X_2_	1.50	1	1.50	7.27	0.0225*
X_1_ X_3_	5.17	1	5.17	25.09	0.0005*
X_2_ X_3_	11.49	1	11.49	55.74	<0.0001*
X_1_^2^	68.54	1	68.54	332.41	<0.0001*
X_2_^2^	0.78	1	0.78	3.78	0.0805
X_3_^2^	0.73	1	0.73	3.53	0.0895
Residual	2.06	10	0.21		
Lack of Fit	1.56	5	0.31	3.13	0.1182
Pure Error	0.50	5	0.10		
Cor Total	196.90	19			

“*Significant values, *df*: Degree of freedom, *F*: Fishers’s function, *P*: Level of significance”.

**Table 8:** The fit summary.

**Sequential Model Sum of Squares**					
**Source**	**Sum of Squares**	***df***	**Mean Square**	***F-*** **value**	***P-*** **value** ***P*** **rob >** ***F***
Linear vs Mean	16.2898	3	5.4299	0.4810	0.7000
Two factors interaction (2FI) vs Linear	18.1659	3	6.0553	0.4846	0.6988
Quadratic vs 2FI	160.3796	3	53.4599	259.2608	< 0.0001*
Residual	1.4894	6	0.2482		
**Lack of Fit Tests**					
**Source**	**Sum of Squares**	***df***	**Mean Square**	***F-*** **value**	***P-*** **value** ***P*** **rob >** ***F***
Linear	180.1078	11	16.3734	163.8444	< 0.0001*
Two factors interaction (2FI)	161.9419	8	20.2427	202.5633	< 0.0001*
Quadratic	1.5623	5	0.3125	3.1268	0.1182
Pure Error	0.4997	5	0.0999		
**Model Summary Statistics**					
**Source**	**Standard deviation**	**R-Squared**	**Adjusted R-Squared**	**Predicted R-Squared**	**PRESS**
Linear	3.3598	0.0827	−0.0893	−0.6167	318.3173
Two factors interaction (2FI)	3.5349	0.1750	−0.2058	−4.2134	1026.4997
Quadratic	0.4541	0.9895	0.9801	0.9494	9.9712

“* Significant values, *df*: degree of freedom”.

With respect to the coefficients (Table 6), interactions between two factors could appear as a synergistic effect (positive coefficient) or an antagonistic effect (negative coefficient). The positive coefficients for X_1_, X_2_, X_3_, X_1_X_3_ (Table 6) indicate that linear effect of X_1_, X_2_, X_3_ and interaction effects for X_1_, X_3_ increase production of cholesterol oxidase, whereas other negative coefficients indicate decrease in the production of cholesterol oxidase.

Table 7 illustrates the analysis of variance (ANOVA) values for the quadratic regression model obtained from FCCD employed in the optimization of cholesterol oxidase production. The *P*-value was calculated to validate the significance of each coefficient and is also necessary for understanding the mutual interactions between the factors. The *P*-values are listed in Table 7. Values of “Prob > *F*” (*P*-values) less than 0.05 indicates the significance of model terms. Values greater than 0.05 indicate the model terms are not significant. The analysis of variance confirms that the model is highly significant as is evident from Fisher’s *F*-test (104.99) and a very low *P*-value < 0.0001. It can be seen that the linear coefficients of cholesterol concentration, incubation time and pH, the interaction between cholesterol concentration, incubation time, interaction between cholesterol concentration, pH and interaction between incubation time, pH and quadratic effect of cholesterol concentration (X_1_) are significant while quadratic effect of incubation time (X_2_) and pH (X_3_) is not significant (*P*-value < 0.05) (Table 7). The probability values of the coefficient indicated that, among the studied three variables pH, the interactions between incubation time and pH and quadratic effect of cholesterol concentration shows maximum *P*-value > 0.0001 demonstrating 99.99% of the model affected by these variables.

As illustrated in Table 8, the statistics of the quadratic model summary showed the highest adjusted, R-squared and predicted R-squared of 0.9801, 0.9895 and 0.9494; respectively and lower standard deviation of 0.4541. The fit summary confirmed the adequacy and the high significance of the quadratic model with a very low *P*-value < 0.0001.

In order to determine the relationship between the production of cholesterol oxidase and the independent variables and to determine the optimal concentration of each component involved in cholesterol oxidase production mainly, concentration of cholesterol, time of incubation and value of pH, the equation of second-order polynomial was obtained to define the predicted cholesterol oxidase production (Y) in terms of the independent variables:

2 $$\begin{array}{ccc}{\bf{Y}} & = & +14.940+0.650{{\rm{X}}}_{1}+0.370{{\rm{X}}}_{2}+1.034{{\rm{X}}}_{3}-0.433{{\rm{X}}}_{1}{{\rm{X}}}_{2}\\  &  & +\,0.804{{\rm{X}}}_{1}{{\rm{X}}}_{3}-1.199{{\rm{X}}}_{2}{{\rm{X}}}_{3}-4.992{{\rm{X}}}_{1}^{2}-0.532{{\rm{X}}}_{2}^{2}-0.515{{\rm{X}}}_{3}^{2}\end{array}$$

Where Y is the predicted activity of cholesterol oxidase and X_1_, X_2_ and X_3_ are concentration of cholesterol, time of incubation and pH; respectively.

### Three dimensional plots

**Figure 4:**
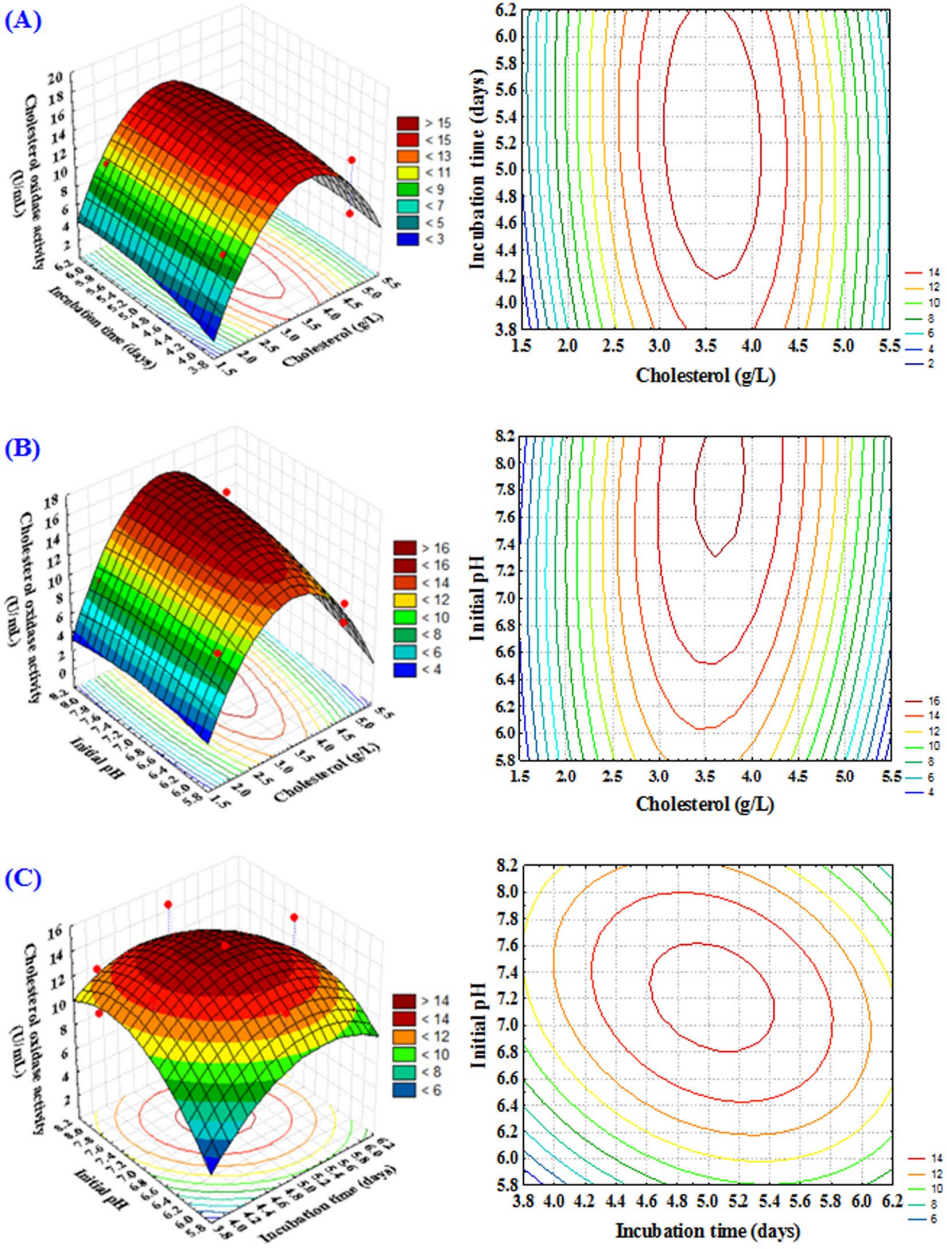
3D response surface and contour plots of the influences of cholesterol concentration (X_1_), time of incubation (X_2_) and pH (X_3_) and their mutual effect on the cholesterol oxidase activity.

The three-dimensional surface graphs and its corresponding contour plots explained the interaction of different studied factors and optimal levels of each factor involved in cholesterol oxidase production. Response plotting curves represented the effect of one fixed variable at its optimum level when the other two factors are varying (Fig. 4A–C). Figure 4A illustrates cholesterol oxidase activity as affected by the concentration of cholesterol (X_1_) and incubation time (X_2_) by keeping the initial pH (X_3_) at the best value. It showed that, when cholesterol concentration increases, production of cholesterol oxidase gradually increases, but increasing cholesterol concentration higher than 3.5 g/L can lead to reduction in cholesterol oxidase production. Also, increasing of incubation time increases the enzyme production and increasing of incubation time above 5 days can lead to reduction in cholesterol oxidase production. Figure 4B showed the effect of cholesterol concentration (X_1_), value of initial pH (X_3_) by keeping incubation time (X_2_) at optimal value on cholesterol oxidase production. In this experiment, moderate values of both cholesterol concentration and initial pH yielded the maximum activity of cholesterol oxidase, so any further increase will result in a gradual decrease in the enzyme production. Furthermore, significant interactions between these variables have greatly helped to increase the activity of cholesterol oxidase. Figure 4C represents the activity of cholesterol oxidase as affected by incubation time (X_2_), value of initial pH (X_3_) by keeping cholesterol concentration (X_1_) at optimum value. In this experiment, the moderate values of both incubation time and initial pH value yielded the maximum activity of cholesterol oxidase. Gradual decrease in cholesterol oxidase activity was seen as a function of any further increase in these variables.

### Validation of the model

The accuracy determination and validation of the generated statistical model and regression equation was accomplished by carrying out an experiment using the optimized conditions, which showed cholesterol oxidase production of 15.074 UmL^−1^. This value of cholesterol oxidase production corresponds very well to the value predicted by the fitted model (predicted response 15.459 UmL^−1^). The close relationship between the predicted and experimental response value was clearly confirmed and demonstrated the accuracy of the model and proving its validity under the tested conditions. The verification showed a high model accuracy degree of 97.51%, indicating the validation of the model.

### The effect of storage period on cholesterol oxidase stability

The effects of pH, temperature and storage (for 7 days at room temperature and 4 °C) on the stability of cholesterol oxidase produced by *Streptomyces aegyptia* NEAE-102 were studied and previously published^46^. The effect of long storage period at −20 °C on cholesterol oxidase stability has been studied. Around 70% of the initial enzyme activity was retained by the enzyme after 8 months of storage at −20 °C, while 19.71% of the initial enzyme activity was observed after storage at −20 °C for 16 months. “The storage life of cholesterol oxidase from *Streptomyces lavendulae* NCIM 2421 was 5 days when kept in a sterile container at room temperature. When stored at 4 °C full enzyme activity is retained for 60 days and had a half life of 105 days^20^. However, the storage life of cholesterol oxidase from *Streptomyces parvus* was 6 months at 4 °C^16^”.

### Effect of cholesterol oxidase on serum cholesterol

*In vitro* effect of cholesterol oxidase on the serum cholesterol was studied and the results revealed significant effect of cholesterol oxidase on the serum cholesterol, the value of cholesterol after incubation of serum cholesterol with the diluted enzyme was 188 mg/dl compared with its value before incubation which was 390 mg/dl. “Cholesterol oxidase has a very critical role in the clinical evaluation of cholesterol levels in serum, HDL, LDL for the assessment of atherosclerotic diseases and other lipid disorders as well as the risk of thrombosis^47^. The normal level of cholesterol in blood of human is less than 200 mg/dl and about ~70% of cholesterol present in lipoproteins is in esterified form. High levels of cholesterol and cholesteryl esters (hypercholesterolemia) are associated with cardiovascular diseases like atherosclerosis and other heart diseases, although lower levels of cholesterol and cholesteryl esters (hypocholesterolemia) may be associated with cancer, depression or respiratory diseases making the determination of cholesterol concentration in serum very important^3^. The risk of Alzheimer’s disease is also related to hypercholesterolemia by involving oxidative stress mechanisms^48^. “Recently, different electrochemical biosensors using the immobilized cholesterol oxidase have been reported for cholesterol determination in serum or food”^49–51^.

### Assessment of *in vitro* cytotoxicity and anticancer activities

**Figure 5:**
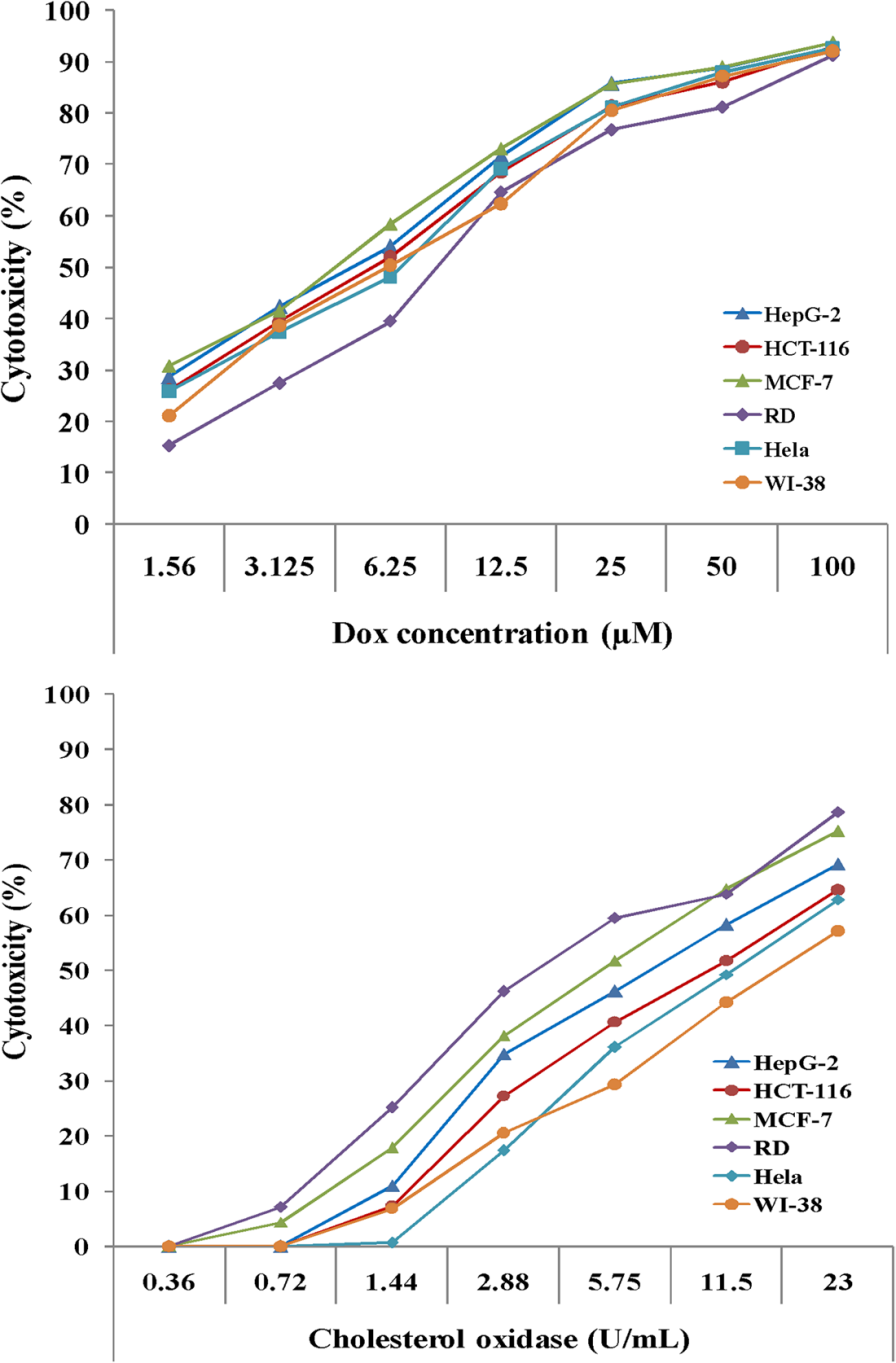
Cytotoxicity of five human tumor cell lines namely; rhabdomyosarcoma (RD), breast cancer (MCF-7), hepatocellular carcinoma (HepG-2), cervical epithelioid carcinoma (Hela), colon carcinoma (HCT-116) and non-cancerous cell line (human lung fibroblast, WI-38).

The purified cholesterol oxidase of *Streptomyces aegyptia* strain NEAE-102 was assessed for its anticancer activity (*in vitro*) using microculture tetrazolium (MTT) assay against five human cancer cell lines namely; rhabdomyosarcoma (RD), breast cancer (MCF-7), hepatocellular carcinoma (HepG-2), cervical epithelioid carcinoma (Hela), colon carcinoma (HCT-116) and non-cancerous cell line (human lung fibroblast, WI-38). The obtained results revealed that the purified cholesterol oxidase of *Streptomyces aegyptia* strain NEAE-102 exhibited a variable degree of inhibitory activity toward the five tested human cancer and normal cell lines (Fig. 5). Cholesterol oxidase at 23 UmL^−1^ inhibited the cell viability of HepG-2, HCT-116, MCF-7, RD, Hela and normal WI-38 cell lines by 69.4, 64.6, 75.3, 78.7, 62.9 and 57.2%; respectively.

The potent cytotoxic activity of cholesterol oxidase against five cancer cell lines and moderate cytotoxicity against normal non-cancerous cell lines shows that cholesterol oxidase can be used as potential natural anticancer. Cholesterol oxidase has the ability to convert membrane cholesterol to cholestenone and block the formation of lipid rafts^52^. “Cholesterol oxidase differs from other cholesterol-depleting agents, in disrupting lipid rafts by displacing cholesterol with cholestenone. Cholesterol oxidase does not attack all cell types because it’s action depend on the microenvironment of membrane cholesterol, like the composition of phospholipid, cholesterol content and ionic strength. So far, despite the fact that cholesterol oxidase has been well known as a destroyer for lipid rafts, few investigations have been conducted to directly determine the effect of cholesterol oxidase on signal transduction in cancer”^13^. Liu *et al*.^13^ reported that cholesterol oxidase from *Bordetella* spp. made the cells of lung cancer to undergo irreversible apoptosis both *in vitro* and *in vivo*.

Table 9 shows the selectivity index of cholesterol oxidase or doxorubicin tested against the cancer cell lines. Treatments of cancer cell lines with cholesterol oxidase (23 UmL^−1^) gave selectivity index ranged from 1.26 to 3.26 which is higher than that of the standard anticancer drug doxorubicin which ranged from 0.73 to 1.45 (Table 9), demonstrating superiority of cholesterol oxidase over the clinically used anticancer doxorubicin and that the cholesterol oxidase has therapeutic potential. The selectivity index of the purified cholesterol oxidase on RD cell line reached 3.26. This value means that the cholesterol oxidase is toxic to the cancer cell line by more than thrice in comparison to the normal cell line. “The selectivity index considered interesting if the value is greater than 3 as reported by Bézivin *et al*.^53^. On the other hand, the selectivity index of the purified cholesterol oxidase on HepG-2, HCT-116, MCF-7 and Hela cell lines reached 1.94, 1.47, 2.56 and 1.26; respectively. Selectivity index value greater than or equal to 2 is an interesting as reported by Suffness and Pezzuto^54^”. These results demonstrated that cholesterol oxidase is a promising anticancer drug.

**Table 9:** The cancer cell selectivity index of the purified cholesterol oxidase of *Streptomyces aegyptia* strain NEAE-102 and doxorubicin on cancerous cells.

Compounds	HepG-2	HCT-116	MCF-7	RD	Hela
**Dox (µM)**					
Cancer cells selectivity index (SI)	1.34	1.15	1.45	0.73	1.08
**Cholesterol oxidase (UmL** ^**−1**^ **)**					
Cancer cells selectivity index (SI)	1.94	1.47	2.56	3.26	1.26

### *In vivo* effect of cholesterol oxidase on solid tumor growth inhibition

**Figure 6:**
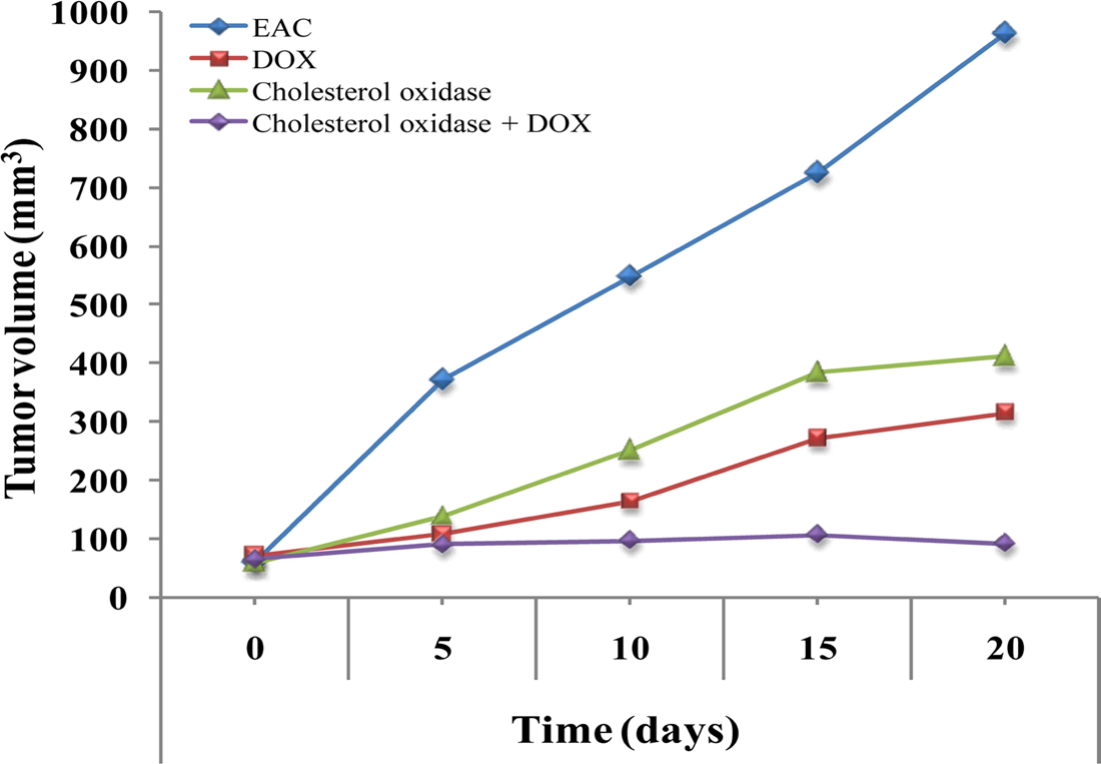
Effect of cholesterol oxidase treatment alone or in combination with doxorubicin on tumor volume of EAC bearing mice revealed cholesterol oxidase-induced apoptosis.

**Figure 7:**
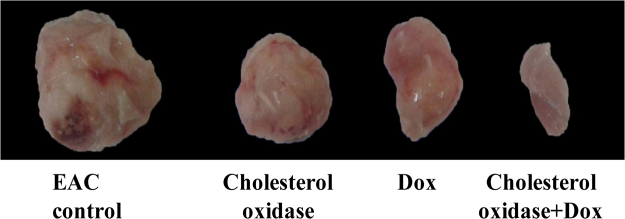
Effect of cholesterol oxidase treatment alone or in combination with doxorubicin on tumor weight of EAC bearing mice revealed cholesterol oxidase-induced apoptosis.

**Figure 8:**
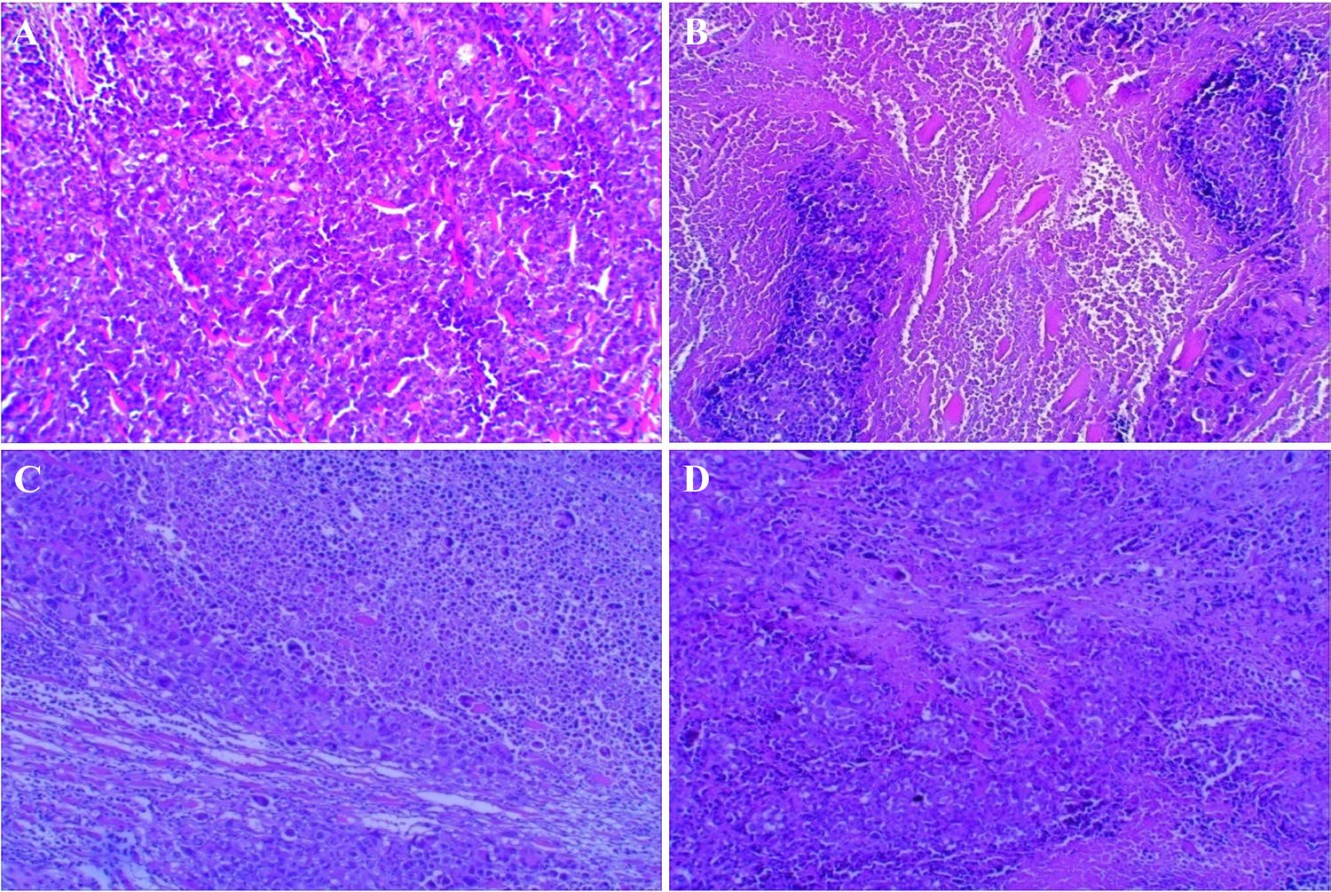
Effect of cholesterol oxidase treatment alone or in combination with doxorubicin on histopathological analysis of tumor sections: microphotographs showing (**A**) untreated mice (control/EAC), (**B**) EAC bearing mice treated with cholesterol oxidase (**C**) EAC bearing mice treated with doxorubicin and (**D**) EAC bearing mice treated with both doxorubicin and cholesterol oxidase.

The use of natural products to treat or prevent the cancer is currently receiving considerable attention^55^. The traditional application of individual drugs fails to treat cancer adequately. Therefore, synergistic combinations of drugs are a promising means of enhancing effectiveness. However, to assess *in vivo* the role of cholesterol oxidase as an effective inducer of apoptosis, the effect of cholesterol oxidase and cholesterol oxidase/ doxorubicin (Dox) in synergistic combination treatment on growth and apoptosis of Ehrlich ascites carcinoma (EAC) solid tumor has been analyzed (Figs 6–8, Table 10). The average tumor volume in the EAC control mice increased from 60.2 mm^3^ to 962.1 mm^3^ after 20 days treatment. Compared with EAC control mice, tumors growth was significantly inhibited in the EAC bearing mice injected with cholesterol oxidase and Dox-treated groups (Fig. 6) by 60.97% and 72.99%; respectively. Moreover, cholesterol oxidase /Dox combination treatment showed a significant tumor growth inhibition (by 97.04%) as compared with both Dox-treated and cholesterol oxidase-treated mice. The quantified weight of tumor lumps in mice injected with cholesterol oxidase, Dox and the combination of cholesterol oxidase/Dox was found to be significantly much smaller compared to EAC control mice. On the other hand, weight of tumor lumps in mice injected with the combination therapy was significantly smaller (0.1 ± 0.01) as compared with the mice injected with Dox (1.7 ± 0.52) or the mice injected with cholesterol oxidase (2.3 ± 0.16) (Fig. 7, Table 10).

**Table 10:** Inhibitory effect of cholesterol oxidase and/or doxorubicin on tumor parameters in EAC bearing mice.

Groups	Tumor weight (g)	Average of tumor volume (mm^3^)		ΔT/ΔC (%)	Inhibition (%)
		0 days	20 days		
EAC	5.8 ± 0.43	60.2	962.1	100.00	0.00
Dox	1.7 ± 0.52	71.3	314.9	27.01	72.99
Cholesterol oxidase	2.3 ± 0.16	58.7	410.7	39.03	60.97
Cholesterol oxidase + Dox	0.1 ± 0.01	64.5	91.2	2.96	97.04

Our results showed that the administration of cholesterol oxidase alone significantly reduced the tumor volume and weight compared to control group. The anti-tumor effect of cholesterol oxidase was near to the therapeutic effect caused by treatment with Dox. The results showed that a significant reduction in tumor in case of the combination of cholesterol oxidase/Dox treatments compared to each treatment alone. These results collectively showed that the combination therapy was able to inhibit tumors growth much more effectively than any of the cholesterol oxidase or Dox alone *in vivo*. Liu *et al*.^13^ reported that “cholesterol oxidase isolated from *Bordetella* species oxidizes membrane cholesterol to 4-cholesten-3-one with the reduction of O_2_ to H_2_O_2_, which results in a decrease of cholesterol content and increase of reactive oxygen species (ROS) levels which leads to cell apoptosis by inactivation of the protein kinase B and extracellular signal-regulated kinase 1/2 pathway as well as activation of caspase-3. Cholesterol oxidase also oxidizes exogenous cholesterol to 4-cholesten-3-one and H_2_O_2_. Therefore, cholesterol addition is not able to prevent the cholesterol oxidase induced apoptosis. These findings suggest that cholesterol oxidase may be a promising therapeutic agent for cancer by targeting membrane cholesterol”^13^.

Histopathological examination of the tumor sections stained with hematoxylin and eosin (Fig. 8) showed the cords of malignant cells, invasion of cellular infiltration and large cancer cells in between muscle fibers in mice that carry untreated tumor (control). Treatment of EAC bearing mice with cholesterol oxidase or doxorubicin showed less tumor invasion, cellular infiltrates, markedly increased apoptotic bodies and moderate mitotic rate. Treatment of EAC bearing mice with both cholesterol oxidase and doxorubicin showed a significant protective synergistic effect on the histopathological architecture.

## Materials and Methods

### Microorganisms and cultural conditions

The *Streptomyces* sp. that used in the present study is a local isolate formerly identified and published as novel species; *Streptomyces aegyptia* NEAE-102^56^. This isolate was preserved on starch-nitrate agar medium slopes^57^. Slopes were incubated at 30 °C for a time of 7 days. The isolate was stored as spore suspension in glycerol (20%, v/v) at −20 °C for the subsequent studies.

### Qualitative screening of actinomycetes isolates for cholesterol oxidase production potentialities using colony staining method

The potentiality of actinomycetes for cholesterol oxidase production was screened on agar plate medium which containing the cholesterol substrate as the sole carbon source according to the method of El-Naggar *et al*.^46^. This experimental medium contained/L: “2 g cholesterol, 2 g KNO_3_, 1 g K_2_HPO_4_, 0.5 g MgSO_4_.7H_2_O, 0.5 g NaCl, 3 g CaCO_3_, 0.01 g FeSO_4_.7H_2_O, 20 g agar and distilled water up to 1 L”. To confirm cholesterol oxidase producing strain, colony staining method was carried out on the grown colonies. Discs of filter papers were dipped in 100 mM potassium phosphate buffer (pH 7.0) containing 0.5% cholesterol; 3000 U/L horseradish peroxidase; 1.7% 4-aminoantipyrine and 6% phenol. “Thereafter, soaked discs were localized on the grown colonies and the plates were incubated at room temperature for 24 h. Cholesterol oxidase activity was assessed by development of pink color surrounding the tested colonies because of the quinoneimine dye formation”^58^.

### Inoculum preparation

The isolate was grown in 250 mL Erlenmeyer flasks containing 100 mL of broth medium consisted of “g/L: (NH_4_)_2_SO_4_ 7.5; cholesterol 2; K_2_HPO_4_ 1; MgSO_4_.7H_2_O 0.5; FeSO_4_.7H_2_O 0.02; NaCl 1; MnSO_4_ 0.008; CaSO_4_ 0.002; ZnSO_4_ 0.002; CaCl_2_ 0.0002; Tween 80 0.05; glucose 12; starch 9; yeast extract 6; peptone 4”^38^. The broth medium was inoculated with 5 discs (9 mm diameter) which taken from stock culture (7 days old) grown on starch nitrate agar medium and incubated at 30 °C with shaking (200 rpm) for 48 h and used for subsequent experiments as inoculum.

### Production of cholesterol oxidase in submerged fermentation

Erlenmeyer flasks (250 mL) containing 100 mL of fermentation medium were inoculated with previously prepared inoculum and incubated at 30–37 °C on a rotatory shaker incubator (150 rpm). After the incubation time, the mycelium was centrifuged at 5000 × *g* for 15 min (at 4 °C). The obtained cell free supernatant after centrifugation was used as crude enzyme for further investigations.

### Cholesterol oxidase activity assessment

Cholesterol oxidase activity was determined spectrophotometry by the modified method of Sasaki *et al*.^59^ which based on generation of hydrogen peroxide during the oxidation reaction of cholesterol. The enzyme reaction was composed of: 0.1 mL crude enzyme, 1 mL cholesterol (3 μM) as the reaction substrate prepared in Triton X-100 (1%), 21 μM of phenol, 1.2 μM 4-aminoantipyrine, 20 U of horseradish peroxidase, 300 μM of potassium phosphate buffer (pH 7) in final reaction volume of 3 mL. The enzyme reaction was incubated with shaking for 10 min at 37 °C. To terminate the reaction, the assay mixture was boiled for 3 min. The developed pink color was measured spectrophotometry at 500 nm. “One unit of the enzymatic activity (U) was defined as the amount of enzyme required to form 1 μmol of H_2_O_2_/min. at 37 °C”.

### Screening of main factors affecting the production of cholesterol oxidase by Plackett–Burman design

The Plackett-Burman statistical design was used to screen and evaluate the influence of significant constituents of medium and the environmental conditions with respect to their main effect on cholesterol oxidase production. In the current work, fifteen independent (assigned) factors were screened with four un assigned variables (commonly referred as dummy variables D_1_, D_2_, D_3_ and D_4_). Based on the Plackett-Burman factorial design, each variable was examined in two levels +1 for the high level and −1 for the low level^60^. The variables selected to be screened by Plackett Burman experiment were cholesterol, peptone, yeast extract, glucose, starch, (NH_4_)_2_SO_4_, K_2_HPO_4_, NaCl, MgSO_4_, FeSO_4_, inoculum size, incubation time, temperature, medium volume and pH. The experiment was carried out in 20 runs to study the influence of the selected factors on cholesterol oxidase production. Plackett–Burman experimental design is based on the first order polynomial equation:

3 $${\boldsymbol{Y}}={{\boldsymbol{\beta }}}_{0}+\sum {{\boldsymbol{\beta }}}_{i}{{\boldsymbol{X}}}_{i}$$

where, Y is the activity of cholesterol oxidase, β_0_ is the coefficient of the model, *β*_*i*_ is the linear coefficient and X_i_ is the independent factors levels.

### Optimization of cholesterol oxidase production by response surface methodology

Face centered central composite design (FCCD) was used in the next step to determine the optimum levels of significant factors for the production of cholesterol oxidase. The face centered central composite design is a statistical experimental design where each factor has three levels (−1, 0, 1) and 6 runs at center point resulting in a total of 20 trials. The second- order polynomial equation was used to calculate the relationship between the independent factors and the response. Considering all the linear terms, square terms and by linear interaction terms, the quadratic regression model can be illustrated as:

4 $${\boldsymbol{Y}}={{\boldsymbol{\beta }}}_{0}+\sum _{i}{{\boldsymbol{\beta }}}_{i}{{\boldsymbol{X}}}_{i}+\sum _{ii}{{\boldsymbol{\beta }}}_{ii}{{\boldsymbol{X}}}_{i}^{2}+\sum _{ij}{{\boldsymbol{\beta }}}_{ij}{{\boldsymbol{X}}}_{i}{{\boldsymbol{X}}}_{j}$$

where “*Y* is the predicted value of cholesterol oxidase production, β_0_ is the regression coefficients, β_i_ is the linear coefficient, β_ij_ is the interaction coefficients, β_ii_ is the quadratic coefficients and X_i_ is independent factors levels”.

### Statistical analysis

“Design Expert software version 7 for Windows was used to perform the experimental designs and statistical analysis and The STATISTICA software version 8 was used to draw 3D surface plots”.

### Effect of cholesterol oxidase on serum cholesterol

Cholesterol oxidase is used for determining cholesterol in different samples as total and esterified cholesterol in serum or blood and in different food samples^7,61^. The serum cholesterol assay is based on an enzyme-mediated reaction which quantifies free cholesterol and cholesterol esters^62^. To estimate serum cholesterol, the initial serum cholesterol value was determined. Then the serum was treated separately with 5 μl of the purified and diluted (1:1) enzyme samples and kept for incubation for 10 min at 37 °C. After completion of the incubation period, the amount of residual cholesterol was estimated.

### Assessment of *in vitro* cytotoxicity and anticancer activities using microculture tetrazolium assay (MTT assay)

The anticancer activities of *Streptomyces aegyptia* NEAE-102 purified cholesterol oxidase were measured *in vitro* on both cancerous (rhabdomyosarcoma (RD), breast cancer (MCF-7), hepatocellular carcinoma (HepG −2), cervical epithelioid carcinoma (Hela), colon carcinoma (HCT-116) and non-cancerous cell line (human lung fibroblast, WI-38). “Which obtained from ATCC via holding company for biological products and vaccines (VACSERA), Cairo, Egypt”. The cell lines mentioned above were used to determine the inhibitory effects of cholesterol oxidase on cell growth using standard 3-(4, 5 dimethythiazol-2-yl)-2, 5-diphenyl tetrazolium bromide (MTT) assay^63^. The cell lines were treated with different concentrations of cholesterol oxidase (23, 11.5, 5.75, 2.875, 1.437, 0.718 and 0.359 UmL^−1^). Doxorubicin was used as a standard anticancer drug for comparison.

### Selectivity index (SI)

In the present study, the degree of selectivity of the cholesterol oxidase is expressed as: SI = IC_50_ of cholesterol oxidase in a normal cell line/IC_50_ of the same cholesterol oxidase in cancer cell line, where IC_50_ is the concentration required to kill 50% of the cell population^64^.

### Cell apoptosis of Ehrlich solid tumor induced by cholesterol oxidase *in vivo*

Ethics statement. “All experimental protocols were approved by Research Ethics Committee, Faculty of science, Mansoura University, Mansoura, Egypt. All the experiments were performed in accordance with the relevant guidelines and regulations. Cholesterol oxidase from *Streptomyces aegyptia* strain NEAE-102 was purified and previously published”^46^. To determine whether cholesterol oxidase induced apoptosis *in vivo*, the effect of cholesterol oxidase on growth and apoptosis of Ehrlich solid tumor was studied.

### Animals

The *in vivo* experiment was performed on “adult Swiss female albino mice with an average body weight of 25–30 g which purchased from Institute of Theodore Bilharz Research, Giza, Egypt, and kept in animal house of Faculty of Pharmacy, Mansoura University, Mansoura, Egypt. Mice were housed in standard size polycarbonate cages under standard laboratory conditions (26 ± 1 °C, 12-h light: 12-h dark cycle) with free access to food and water *ad libitum*”.

### Ehrlich ascites carcinoma cell line

The Ehrlich ascites carcinoma (EAC) cell line was obtained from the National Cancer Institute (NCI), Cairo University, Cairo, Egypt.

### Ehrlich solid tumor model

Mice were injected subcutaneously into the right hind limb (thigh) of all animals, each with 5 × 10^5^ cells, to form solid tumor. Five days post-tumor inoculation when the solid tumor volumes reached approximately 50–100 mm^3^ (day 0), the mice were randomly divided into four groups comprising five animals in each group. Group I served as tumor control injected with EAC; group II represents EAC bearing mice injected in tumor with cholesterol oxidase alone (3 U, q^3^ days); group III represents EAC bearing mice injected with the anti-cancer doxorubicin (Dox) alone (2 mg kg 1 day); group IV represents EAC bearing mice injected with cholesterol oxidase in combination with Dox.

Tumor volumes were measured from the fifth day of tumor induction and before the beginning of the treatment (day 0) and were carried out every five days for a period of 20 days. “Volume of solid tumor was monitored by vernier caliper and was calculated using the following formula: V = (L × S^2^) × 0.5^65^, where (L) is the longest diameter of tumor and (S) is its shortest perpendicular diameter. Anti-tumor effectiveness was evaluated by the estimation of the average change of tumor volume in the treatment group (ΔT) and the average change of tumor volume in the control EAC bearing mice (ΔC). The degree of tumor growth was calculated as ΔT/ΔC × 100 which was then subtracted from 100% to estimate the percentage (%) of tumor growth inhibition”^66^. On the day 21 of treatment process (at the end of the experiment), mice were sacrificed and tumors lumps were removed, weighed and preserved for further histopathological analysis in buffered formalin solution. Five–micrometer sections were stained with hematoxylin and eosin. The slides were examined for histopathological changes using light microscopy.

## Conclusion

The present study involved the use of statistical experimental designs to optimize nutritional and environmental variables for production of cholesterol oxidase from *Streptomyces aegyptia* strain NEAE-102. Three variables which are cholesterol concentration, incubation time and pH were identified by the experiment of Plackett-Burman design as significant for cholesterol oxidase production. These variables were further optimized using a face centered central composite design. The maximum level of cholesterol oxidase activity (15.631 UmL^−1^) was produced when the fermentation medium variables were set as follows: cholesterol concentration 3 g/L, incubation time 5 days, pH 6. The methodology as a whole was proved to be adequate for optimization of medium components for obtaining a therapeutically valuable product as cholesterol oxidase. Treatments of cancer cell lines with cholesterol oxidase (23 UmL^−1^) gave selectivity index ranged from 1.26 to 3.26 which is higher than that of the standard anticancer drug doxorubicin which ranged from 0.73 to 1.45, demonstrating superiority of cholesterol oxidase over the clinically used anticancer doxorubicin and that the cholesterol oxidase can be used as potential natural anticancer. *In vivo* study, the results showed that the combination therapy was able to inhibit tumors growth much more effectively than either cholesterol oxidase or Dox treated groups alone.
